# Comparative Pathogenomics of Bacteria Causing Infectious Diseases in Fish

**DOI:** 10.1155/2012/457264

**Published:** 2012-05-22

**Authors:** Ponnerassery S. Sudheesh, Aliya Al-Ghabshi, Nashwa Al-Mazrooei, Saoud Al-Habsi

**Affiliations:** Microbiology Laboratory, Fishery Quality Control Center, Ministry of Agriculture and Fisheries Wealth, P.O. Box 427, 100 Muscat, Oman

## Abstract

Fish living in the wild as well as reared in the aquaculture facilities are susceptible to infectious diseases caused by a phylogenetically diverse collection of bacterial pathogens. Control and treatment options using vaccines and drugs are either inadequate, inefficient, or impracticable. The classical approach in studying fish bacterial pathogens has been looking at individual or few virulence factors. Recently, genome sequencing of a number of bacterial fish pathogens has tremendously increased our understanding of the biology, host adaptation, and virulence factors of these important pathogens. This paper attempts to compile the scattered literature on genome sequence information of fish pathogenic bacteria published and available to date. The genome sequencing has uncovered several complex adaptive evolutionary strategies mediated by horizontal gene transfer, insertion sequence elements, mutations and prophage sequences operating in fish pathogens, and how their genomes evolved from generalist environmental strains to highly virulent obligatory pathogens. In addition, the comparative genomics has allowed the identification of unique pathogen-specific gene clusters. The paper focuses on the comparative analysis of the virulogenomes of important fish bacterial pathogens, and the genes involved in their evolutionary adaptation to different ecological niches. The paper also proposes some new directions on finding novel vaccine and chemotherapeutic targets in the genomes of bacterial pathogens of fish.

## 1. Introduction

Genome sequencing has provided us with powerful insights into the genetic makeup of the microbial world. The microbial genomics today has progressed from the long drawnout individual genome sequencing projects in the past to a level of technological advancement, where sequencing and comparing the genomes of several strains of a single pathogen is accomplished in a very short period of time [1, 2]. We are currently passing through a period of explosive developments in the field and an overwhelming glut in the genome sequence data of microorganisms. To date, over 1800 microbial genomes have been published and the sequencing of more than 5200 microbial genome are in different stages of completion (http://www.ncbi.nlm.nih.gov/genomes/lproks.cgi).

The genomics information has categorically disproved the earlier thinking that microbial genomes are static and has demonstrated that genomic evolutionary processes are much more flexible and dynamic than previously thought. This has led to the emergence of new ideas such as “uprooting the tree of life” and the concept of “horizontal genomics” [3–8]. This new thinking about microbial genome evolution has emerged from the observations of lineage-specific genome reduction and horizontal gene transfer (HGT), frequently occurring in bacterial genomes. Increasingly, genome sequencing projects have identified an unexpected level of diversity among bacteria, which can often be linked to recombination and gene transfer between a variety of prokaryotic organisms.

There is large variation in size and content of bacterial genomes between different genera and species, and also among strains of the same species. Known genome sizes of bacteria range from under 0.6 to 10 megabases (Mb). The smallest bacterial genomes reported are for the mycoplasmas and related bacteria, with sizes as low as 530 kilobases [9]. It has been emphasized that the adaptive capability (“versatility”) of bacteria directly correlates with genome size [10].

Genome sequencing of bacterial pathogens has produced exciting information on evolutionary relationships between pathogenic and nonpathogenic species and has demonstrated how each has developed special adaptations advantageous for each of their unique infectious lifestyles. In the longer term, an understanding of their genome and biology will enable scientists to design means of disrupting their infectious lifestyles.

The genomes of bacteria are made up of circular or linear chromosomes, extrachromosomal linear or circular plasmids as well as different combinations of these molecules. The functionally related genes are clustered together in very close proximity to each other, and those genes located on the “core” part of the chromosome present a relatively uniform G+C content and a specific codon usage. Closely related bacteria generally have very similar genomes [11].

**Figure 1:**
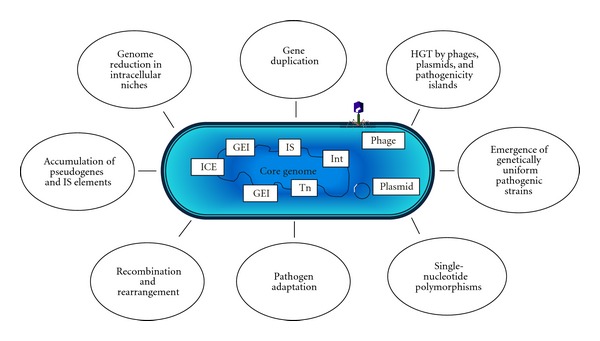
Major factors responsible for the pathogenomic evolution of bacteria (modified from [14, 15]; HGT: horizontal gene transfer, GEIs: genomic islands, ICEs: integrative conjugative elements, Int: integrons, Tn: conjugative transposons, IS: IS elements.

The stability and integrity of the “core” sequences of the genome, however, is often interrupted by the presence of DNA fragments with a G+C content and a codon usage markedly different from those of the “core” genome. The “flexible” gene pool or the so-called “mobilome” [12], is created by the acquisition of strain-specific “assortments” of genetic information mainly represented by mobile genetic elements (MGE), such as plasmids, bacteriophages, genomic/pathogenicity islands (GEIs/PAIs), integrons, IS elements (ISEs), and transposons (see Figure 1). The flexible genes scattered in the genome provide the microbes with an additional repertoire of arsenal, for example, resistance to antibiotics, production of toxic compounds as well as other virulence factors [13].

A fundamental question in biology is to define the minimum number of genes or functions to support cellular life. The size of bacterial genomes is primarily the result of two counteracting processes: the acquisition of new genes by gene duplication or by horizontal gene transfer; the deletion of nonessential genes. Genomic flux created by these gains and losses of genetic information can substantially alter gene content. This process drives divergence of bacterial species and eventually adaptation to new ecological niches [16].

Bacterial pathogens are a major cause of infectious diseases and mortality in wild fish stocks and fish reared in confined conditions. Disease problems constitute the largest single cause of economic losses in aquaculture [17]. Concurrent with the rapid growth and intensification of aquaculture, increased use of water bodies, pollution, globalization, and transboundary movement of aquatic fauna, the list of new pathogenic bacterial species isolated from fish has been steadily increasing [18]. In addition, the virulence and host range of existing pathogens has also been increasing, posing considerable challenge to fish health researchers, who are actively looking for more efficient vaccines and therapeutic drugs to combat bacterial fish diseases. The current treatment methods are ineffective and have many practical difficulties.

At the level of host-pathogen interaction, there is considerable pressure on pathogens to adapt to the harsh host environment as well as to adapt and evolve along with the ever changing external environment. The interplay between the host and the pathogen is a complex one, each driven by the need to secure the success of the species. Adaptations by one partner to exploit new environments will often stimulate the other to modify its characteristics to take advantage of the change. As a consequence of this cycle of interaction created by changing environments, new strains of pathogen will evolve. Over time, these strains may emerge as new species with characteristic disease symptoms.

The use of antibiotics to control fish diseases has met with limited success and has the potential danger of antibiotic resistance development in aquatic bacteria (World Health Organization antimicrobial resistance fact sheet 194, http://www.who.int/inf-fs/en/fact194.html) [19]. As aquaculture is one of the fastest growing food production industries in the world, demand for sustainable ways of combating fish diseases is gaining significance. There is tremendous scope for developing novel vaccines and therapeutic drugs against bacterial fish pathogens.

Genomic evolution and adaptive strategies of bacterial fish pathogens are poorly understood and lags far behind that of human and terrestrial animal pathogens. A detailed knowledge of the genome sequences of bacterial fish pathogens and how the genomes of the pathogenic species or strains evolved from nonpathogenic ancestors or counterparts will help us better understand their pathogenicity mechanisms and strategies of host adaptations. This information will help identifying novel vaccine and drug targets in the genomes of pathogens.

Recently, genome sequencing of a number of bacteria pathogenic to fish and other aquatic organisms have been completed. The genome sequence and genome characteristics of important bacterial fish pathogens completed and published to date are summarized in Table 1.

The main aim of this paper is to put together and summarize the scattered genome sequencing information on important bacterial fish pathogens available in the literature to date. We sincerely believe that this paper will provide a genomic perspective on the adaptive evolutionary strategies of bacterial fish pathogens in different ecological niches and will help better understand the virulence mechanisms and pathogenesis of infections. It is hoped that this will lead to finding the most appropriate vaccine and therapeutic drug targets in the genomes and developing efficient control and treatment methods for fish diseases.

## 2. Bacterial Pathogens of Fish

Although pathogenic species representing majority of existing bacterial taxa have been implicated in fish diseases, only a relatively small number of pathogens are responsible for important economic losses in cultured fish worldwide. Major bacterial pathogens responsible for infectious disease outbreaks in different species of fish are listed in Table 2. Major groups of bacteria causing infectious diseases in fish and the important genome characteristics of these bacteria are described in the following sections. 

## 3. *Vibrios*

Bacteria in the genus *Vibrio* are mainly pathogenic to marine and brackish water fish. However, they are occasionally reported in freshwater species as well [20, 21]. The distribution of vibriosis is worldwide and causes great economic loss to the aquaculture industry [22]. Vibriosis, one of the major bacterial diseases affecting fish, bivalves, and crustaceans, is mainly caused by pathogenic species such as *Vibrio anguillarum*, *V. harveyii *(Syn. *V. carchariae*), *V. ordalii*, and *Aliivibrio salmonicida* (formerly *Vibrio salmonicida*) [23, 24].

Other species such as *V. vulnificus* [25, 26]* and Moritella viscosa *(formerly* Vibrio viscosus*) [27] have been implicated in fish diseases such as septicemia and winter ulcer, respectively; more pathogenic species have been isolated frequently and reported in the literature [28].

Genome sequences of four major fish pathogenic vibrios,* V. anguillarum, V. ordalii, Aliivibrio salmonicida, and V.vulnificus *have been completed and published [29–31]. Generally, they have two chromosomes, one larger and one smaller. The majority of genes that encode cell functions and pathogenic factors are located in the large one. The small chromosome usually contains genes for environmental adaptation.

*Vibrio anguillarum *is the most studied aetiological agent of vibriosis [32]. *V. anguillarum *typically causes a hemorrhagic septicemia. The O1 and O2 serotypes are the virulent strains frequently isolated from diseased fish [33, 34]. Many O1 serotype strains harbor 65 kb pJM1-type plasmids, which carry the siderophore anguibactin biosynthesis and transport genes, a main virulence factor of *V. anguillarum*, while one of the O1 serotype strains and other serotypes, such as all of the O2 strains, are plasmidless [28, 35, 36]. The O1 serotype strains cause disease in salmonid fish, whereas O2 *β* strains are usually isolated from cod and other nonsalmonids [28, 32].

*Vibrio ordalii* is a very close relative of *V. anguillarum* [37] and was previously recognized as *V. anguillarum* biotype 2. Vibriosis caused by these two species are strikingly different based on histological evidences [38]. *V. anguillarum* has a special affinity for blood and loose connective tissue, whereas *V. ordali* is mostly present as aggregates in skeletal and cardiac muscles. *V. ordalii* has a lesser affinity for blood and develops bacteremia only at late stages of disease.

Genomic sequences of three different strains of *V. anguillarum* (the strain 775 containing plasmid pJM1, serotype O1 strain 96F, and plasmidless serotype O2 *β* strain RV22) and *V. ordali* have recently been published [31]. The pJM1 plasmid in the strain 775 contains 65 genes including the anguibactin biosynthesis and transport genes that are unique for the strain.

*V. anguillarum *775 contains more transposase genes (about 53) than 96F (about 23), RV22 (about 42), and *V. ordalii *(about 18).

The genome comparison of *V. anguillarum* serotypes has revealed some interesting differences in the genomic composition, indicating horizontal acquisition of virulence genes and the evolution of different potential virulence mechanisms among the closely related serotypes [31]. The *V. anguillarum *96F strain has a type III secretion system 2 (T3SS2) cluster, which is absent in the 775 strain. The T3SS2 genes are highly conserved with other T3SS2 genes reported in *V. parahaemolyticus, V. cholera*, and *V. mimicus *[39–41]. In the 775 strain, three transposase genes are present at the T3SS2 chromosomal location, one of which probably originated from the pJM1, indicating that the gene cluster is inactivated by a transposition, deletion, or inversion event [31]. The 775 strain also contains 10 genomic islands including integrase, transposase, and some novel sequences conferring genomic plasticity to adapt to specific ecological niches.

The strain RV22 genome contains the toxin-antitoxin systems, and genes encoding the accessory *V. cholerae *enterotoxin (Ace) and the *Zonula occludens *toxin (Zot), which is not present in the 775 strain. The yersiniabactin-like siderophore cluster, which is highly conserved in many *Vibrio *species and *Photobacterium damselae *subspecies piscicida [42], is present in strain RV22 and *V. ordalii*.

A striking feature of *V. ordali* genome is its significant reduction in size (3.4 Mb) compared to the *V. anguillarum* strains 775 (4.1 Mb), 96F (4.0 Mb), and RV22 (4.0 Mb).* V. ordali* lacks the ABC transporter genes, the type VI secretion systems, and the gene for microbial collagenase. The Syp biofilm formation cluster, which is conserved in many *Vibrio* species such as *V. fischeri*, *V. vulnificus*, and *V. parahaemolyticus* [43, 44], is present only in *V. ordalii*. Thus, it is probable that the transition of *V. anguillarum* to* V. ordalii* is mediated by genome reductive evolution to become an endosymbiotic organism; *V. ordali* has the smallest genome of all vibrios.

*Vibrio vulnificus *includes three distinct biotypes. Biotype 1 strains cause human disease, while biotype 2 infects primarily eels, and biotype 3 infections has been associated with persons handling Tilapia, although the source and reservoir of biotype 3 have yet to be identified [45]. In another classification the terms clade 1 and clade 2 are used based on the multilocus sequence typing (MLST) [46]. Biotype 1 strains are present in both clades, whereas biotype 2 strains are present only in clade 1, and biotype 3 strains appear to be a hybrid between clades 1 and 2. Clade 1 strains are most often isolated from environmental samples, while clade 2 strains are mostly associated with human disease and are considered more virulent. Recent comparative genomic analysis of these biotypes or clades has clearly differentiated them based on the possession of an array of clade-specific unique genes including the presence of a virulence-associated genomic island XII in the highly virulent strains [30].

*Aliivibrio salmonicida* (formerly *Vibrio salmonicida*) causes coldwater vibriosis in marine fish such as farmed Atlantic salmon (*Salmo salar*), sea-farmed rainbow trout (*Oncorhynchus mykiss*), and captive Atlantic cod (*Gadus morhua*) [47]. The Gram-negative bacterium causes tissue degradation, hemolysis, and sepsis *in vivo*. Genome sequencing of *Aliivibrio salmonicida* has revealed a mosaic structure of the genome caused by large intrachromosomal rearrangements, gene acquisition, deletion, and duplication of DNA within the chromosomes and between the chromosomes and the plasmids [29].

The genome has many genes that appear to be recently acquired by HGT, and large sections of over 300 coding sequences (CDS) are disrupted by IS elements or contain point mutations causing frame shifts or premature stop codons [29]. The genomic islands (GIs) identified in the bacteria include major virulence-related genes encoding T6SS and Flp-type pilus and genes that appear to provide new functions to the bacteria. The Tad system has been proposed to represent a new subtype of T2SS and is essential for biofilm formation, colonization, and pathogenesis [48].

The genome analysis has unequivocally confirmed that *Aliivibrio salmonicida* has undergone extensive rearrangement of its genome by losing massive functional genes and acquiring new genes and become host-restricted, allowing the pathogen to adapt to new niches. IS expansion has been related to genome reduction in the evolution and emergence of pathogenicity [49], and accumulation of pseudogenes has been described for several other host restricted pathogens [50, 51].

## 4. *Aeromonads*

*Aeromonas hydrophila* and other motile aeromonads are among the most common bacteria in a variety of aquatic environments worldwide, including bottled water, chlorinated water, well water, sewage, and heavily polluted waters, and are frequently associated with severe disease among cultured and feral fishes, amphibians, reptiles, and birds [52]. Aeromonads are also considered serious emerging pathogens of human beings [53]. Determination of the etiology of diseases involving aeromonad infections has been complicated by the genetic, biochemical, and antigenic heterogeneity of members of this group.

The genus *Aeromonas *has been conveniently divided into a group of nonmotile, psychrophilic species, prominently represented by *Aeromonas salmonicida*, which is an obligate fish pathogen and a second group of mostly human pathogenic, motile, and mesophilic species including *A. hydrophila*.

Genome sequencing of *A. hydrophila *ATCC 7966^T^,* A. salmonicida *subsp. salmonicida A449, *A. veronii* strain B565, and *A. caviae* [54–57] has helped in resolving their taxonomic confusion and has brought new insights into the way these bacteria adapt to a myriad of ecological niches, their host adaptive evolution and virulence mechanisms.

*Aeromonas salmonicida*, the causative agent of furunculosis in salmonid and nonsalmonid fish, is a non-motile, Gram-negative bacterium; furunculosis is an important disease in wild and cultured stocks of fish inflicting heavy losses to aquaculture industry worldwide [58, 59]. *A. hydrophila *causes a septicemic disease in fish known variously as “motile aeromonas septicemia” (MAS), “hemorrhagic septicemia,” “ulcer disease,” or “red-sore disease” [60]. The disease caused by this bacterium primarily affects freshwater fish such as catfish, several species of bass, and many species of tropical or ornamental fish. *A. veronii *is the causative agent of bacterial hemorrhagic septicemia in fish and is becoming a major economic problem in the fish-farming industry [23].

Genome sequencing of the fish pathogen *A. salmonicida* A449 has confirmed the presence of fully functional genes for a type III secretion system (T3SS) that has been shown to be required for virulence in *A. salmonicida *[61], and genes for a type VI secretion system (T6SS), which is disrupted by an IS element [55]. The ancestral state of the T3SS in *A. salmonicida* A449 is ambiguous because of the absence of the genes in* A. hydrophila *ATCC 7966^T^, while other *A. hydrophila *strains carry T3SS operons on the chromosome [62]. The genome contains a multitude of virulence-related genes including several types of adhesins (e.g., surface layer, flagella, and pili), toxin genes (aerolysin, hemolysin, repeats in toxin (RTX) protein, and cytolytic delta-endotoxin), secreted enzymes (protease, phospholipase, nuclease, amylase, pullulanase, and chitinase), antibiotic resistance genes (*tetA*, **β*-lactamase* gene, and efflux pumps), and genes involved in iron acquisition and quorum sensing.

Most of the above genes are present in *A. hydrophila *ATCC 7966^T^ genome and an expansion of gene families (paralogs) of ABC transporters, two-component signal transduction systems (TCSs), transcriptional regulators, FeS cluster-binding proteins involved in energy transduction at the membrane, and methyl-accepting chemotaxis proteins (MCPs). Interestingly, transposase, resolvase, or insertion sequence element sequences were not discovered in the *A. hydrophila *ATCC 7966^T^ genome, whereas these have been identified in *A. salmonicida* and *A. caviae* genomes. *A. salmonicida *possesses 88 copies of 10 different IS elements whereas *A. caviae *Ae398 has only five different IS elements, and *A. hydrophila *completely lacks IS elements.

Although *A. hydrophila *ATCC 7966^T^ has been demonstrated to be the second most virulent species among *Aeromonas* [63], a very important virulence determinant, T3SS, which is present in *A. salmonicida* A449 is strikingly absent in *A. hydrophila *ATCC 7966^T^ genome. *A. caviae *contains many putative virulence genes, including those encoding a type 2 secretion system, an RTX toxin, and polar flagella.

The genome of *A. veronii* strain B565 contains some putative virulence factors, such as chitinase, RTX protein, adhesion factor, flagella, and mannose-sensitive hemagglutinin (MSHA), all of which are shared with *A. hydrophila *ATCC 7966^T^ and *A. salmonicida *A449. On the other hand, 346 genes including some important putative virulence factors such as hemolysins and the type III secretion protein, which are shared by the latter two species are absent in *A. veronii* strain B565.

Many unique genes in *A. hydrophila *ATCC 7966^T^ and *A. salmonicida *A449 are virulence genes and often form large clusters, such as the *rtx *cluster in ATCC 7966^T^ and the flagellar gene cluster in A449, or are involved in mobile elements such as phages and transposons, highlighting their lateral transfer history [56].

The *A. hydrophila *ATCC 7966^T^ and *A. salmonicida *A449 genomes appear to be very closely related, encoding similar number of proteins with only 9% difference in gene content. However, there are many transposons, phage-related genes, and unique CDS in *A. salmonicida *A449 genome that are different from *A. hydrophila *ATCC 7966^T^ sequences, showing their distinct lineages and adaptive evolution that occurred while segregating into different species of the genus.

In sharp contrast to *A. hydrophila *ATCC 7966^T^ genome, the *A. salmonicida *A449 genome is characterized by the presence of large numbers of several different types of IS elements in multiple copies, with more than 20 genes being interrupted by IS elements.* A. hydrophila *ATCC 7966^T^ genome has no IS elements.

There is a higher tendency for genomic reduction in *A. salmonicida *A449 with the formation of many pseudogenes, and *A. hydrophila *ATCC 7966^T^ has only seven pseudogenes. The formation of pseudogenes has resulted in the loss of function of many genes including flagella and type IV pili, transcriptional regulators, genes encoding carbohydrate synthesis, and modification enzymes and genes for basic metabolic pathways, which are some characteristic features of pathogenomic evolution.

Thus, *A. salmonicida *A449 appears to have evolved much faster than* A. hydrophila *ATCC 7966^T^ through genetic rearrangements, genomic reduction, and HGT from common ancestral lineages by acquiring and forming multiple plasmids, prophages, a battery of IS elements, pseudogenes, and several individual genes and operons.

## 5. *Flavobacterium*

The genus *Flavobacterium *includes over 30 species of which *Flavobacterium psychrophilum*, *F. branchiophilum*, and* F*. *columnare* are important disease agents for salmonids, catfish, and other cultured species [64, 65]. Flavobacteria are significant as they are ubiquitous in the soil, freshwater, and marine environments and are noted for their novel gliding motility and ability to degrade polymeric organic matter such as hydrocarbons [66].

*F. psychrophilum* is the etiological agent of bacterial coldwater disease (BCWD). It is a serious fish pathogen causing substantial economic losses and rearing difficulties to both commercial and conservation aquaculture. *F. psychrophilum *infections are found throughout the world. Juvenile rainbow trout and coho salmon are particularly susceptible to BCWD. However, *F. psychrophilum* infections have been reported in a wide range of hosts, Anguilla* japonica*, *A. anguilla*, *Cyprinus carpio*, *Carassius carassius, Tinca tinca*, *Plecoglossus altivelis*, *Perca fluviatilis*, and *Rutilus rutilus* [64, 67]. Fry and fingerlings with BCWD often have skin ulcerations on the peduncle, anterior to the dorsal fin, at the anus, or on the lower jaw and mortalities can go up to 70% [68].

*F. branchiophilum *is the causative organism of bacterial gill disease (BGD) in several parts of the world [69]. This disease is characterized by explosive morbidity and mortality rates attributable to massive bacterial colonization of gill lamellar surfaces and progressive branchial pathology stemming from high rates of lamellar epithelial necrosis [70].

*F*. *columnare* (formerly *Cytophaga columnaris*; *Flexibacter columnaris*) is the causative agent of columnaris disease of salmonids and other fishes in commercial aquaculture, the ornamental fish industry, and wild fish populations worldwide [71]. Classically, during outbreaks, its morbidity and mortality rates escalate more gradually than for BGD. Additionally, unlike the pattern of necrosis in BGD, fish with columnaris will have severe necrosis of all parts of the gill as the bacterium invades inwardly [72].

The taxonomy of the three species was initially based on phenotypic characteristics and has been revised several times during the years. The latest classification based on G+C content, DNA-ribosomal ribonucleic acid (rRNA) hybridisation, and fatty acid and protein profiles, has confirmed that all the three species now belong to the phylum/division *Cytophaga-Flavobacterium-Bacteroides*, family *Flavobacteriaceae*, and genus *Flavobacterium *[73].

The whole genome sequences of *F. psychrophilum* and *F. branchiophilum* have been published [74, 75]. The *F. columnare* genome sequence is yet to be completed and published [76].

Prominent features of *F. psychrophilum* infection include the strong adhesion to fish epithelial tissues followed by gliding motility, rapid and mass tissue destruction, and severe muscle tissue ulcerations. Hence, the identification of multiple genes encoding secreted proteases, adhesins, and gliding motility (*gld*) genes in *F. psychrophilum* genome indicates their possible involvement in the virulence of the pathogen. However, the gene sequence of a secreted collagenase was disrupted by an insertion sequence of the IS256 family in several strains isolated from rainbow trout [74] indicating the clonal dissemination of strains containing the disrupted gene. The *F. psychrophilum* seems to have horizontally acquired virulence associated genes from other unrelated bacteria. It has a hemolysin similar to the toxin VAH5, which is a virulence factor in *Vibrio anguillarum* [77]. It also has a gene encoding a protein that is similar to domains 1–3 of thiol-activated cytolysin family of pore-forming toxins (TACYs), which has been implicated in the pathogenicity of several Gram-positive bacteria [78]. Interestingly, *F. psychrophilum *lacks the type III and IV secretion systems usually present in Gram-negative pathogens; but, it has genes encoding PorT and PorR proteins, which are involved in transport and anchoring of virulence factors of the bacteria [79, 80]. In addition, the *F. psychrophilum* genome contains a large repertoire of genes involved in aerobic respiration, psychrotolerance, and stress response.

The sequencing of *F. branchiophilum* genome has revealed the existence of virulence mechanisms distinctly different from the closest species, *F. psychrophilum*. The *F. branchiophilum* genome has the first cholera-like toxin in a nonproteobacteria and an array of adhesins. A comparative analysis of its genome with genomes of other *Flavobacterium* species revealed a smaller genome size, large differences in chromosome organization, and fewer rRNA and tRNA genes, fitting with its more fastidious growth. In addition, identification of certain virulence factors, genomic islands, and CRISPR (clustered regularly interspaced short palindromic repeats) systems points to the adaptive evolution of *F. branchiophilum* by horizontal acquisition of genes.

## 6. *Edwardsiella*

The genus *Edwardsiella* belongs to subgroup 3 of *γ*-proteobacteria, encompassing a group of Gram-negative enteric bacteria pathogenic to a variety of animals [81]. Two very closely related species, *Edwardsiella tarda *and *E. ictaluri *are important fish pathogens. Both are Gram-negative motile rods that are cytochrome oxidase negative and ferment glucose with production of acid and gas. The two species can be differentiated biochemically in that *E. tarda *produces both indol and hydrogen sulfide, whereas *E. ictaluri *produces neither. Moreover, the two species do not cross-react serologically. *E. tarda *has been isolated from many warm water fishes and some coldwater fishes, whereas *E. ictaluri *has been isolated only from a few species of warm water fishes (Table 2). Additionally, *E. tarda* causes disease in such other animals as marine mammals, pigs, turtles, alligators, ostriches, skunks, and snakes [81]. It has also occasionally infected humans [82, 83]. In contrast, *E. ictaluri *is limited to fish, and survivors of epizootics probably become carriers. The geographic range of *E. tarda *is worldwide, whereas that of *E. ictaluri *is still confined to the catfish growing areas in the United States [84].

*E. tarda *causes a disease condition in fish called systemic hemorrhagic septicemia with swelling skin lesions as well as ulcer and necrosis in internal organs such as liver, kidney, spleen, and musculature [85]. It has the ability of invading and multiplying in epithelial cells and macrophages in order to subvert the host immune system and to survive in the fish [86].

*E. ictaluri* is the causative agent of enteric septicemia of catfish (ESC), a major disease affecting the catfish industry. The disease can manifest as an acute form that is characterized by hemorrhagic enteritis and septicemia and a chronic disease that is characterized by meningoencephalitis [87]. Gross external symptoms include hemorrhages on the body, especially around the mouth and fins. Other signs include pale gills, exophthalmia, and small ulcerations on the body [84].

The whole genome sequencing of the two species has recently been completed and published allowing comparative genomic analysis of these very important fish pathogens [88, 89]. The genome sequencing of the two closely related species *E. tarda* and *E. ictaluri* has revealed a high level of genomic plasticity with a high content of mobile genetic elements, IS elements, genomic islands, phage-like products, integrases, or recombinases. *E. ictaluri* displays high biochemical homogeneity with only one serotype, but possess many IS elements in the genome. In addition, highly variable G+C content and a large quantity of variable number of tandem repeats (VNTRs) or direct repeat sequences were identified in the *E. tarda* genome indicating the rapid genomic evolution undergoing in the species [88]. An interesting feature is the identification of insertion sequence IS *Saen1* of *Salmonella enterica* serovar Enteritidis [90] in both *E. tarda* EIB202 and *E. ictaluri* 93–146 genomes. Conversely, the difference in genomic islands among the three species may partially explain their rapid evolutionary changes and diverging lineage from a common ancestor.

The *E. tarda* genome has a gene cluster sharing high similarities to the *pvsABCDE-psuA-pvuA* operon, which encodes the proteins for the synthesis and utilization of vibrioferrin, an unusual type of siderophore requiring nonribosomal peptide synthetase (NRPS) independent synthetases (NIS) and usually mediating the iron uptake systems in *V. parahaemolyticus *and* V. alginolyticus* [91, 92]. But *E. ictaluri* genome lacks siderophore biosynthesis genes, even though it possesses heme binding/transport genes.

*E. tarda* genome is smaller than that of *E. ictaluri* and other sequenced genomes of *Enterobacteriaceae*, justifying the hypothesis that *E. tarda *may not be present as a free living microorganism in natural waters but multiply intracellularly in protozoans and transmitted to fish, reptile, and other animals or humans [81].

The *E. tarda* and *E. ictaluri* genomes have a multitude of virulence factors including P pilus, type 1 fimbriae, nonfimbrial adhesins, invasins and hemagglutinins and various secretion pathways including sec-dependent transport system, the components of the main terminal branch of the general secretory pathway (GSP), the signal recognition particle (SRP), and the sec-independent twin arginine transport (Tat), T1SS, TTSS, and T6SS indicating their evolutionary fitness and ability to adapt to a variety of demanding ecological niches and harsh host intracellular environments.

## 7. *Yersinia ruckeri*

*Yersinia ruckeri*, the causal agent of enteric redmouth (ERM) disease, which is a systemic bacterial infection of fishes, but is principally known for its occurrence in rainbow trout, *Salmo gairdneri *[93].* Y. ruckeri *was initially isolated from rainbow trout in the Hagerman Valley, Idaho, USA, in the 1950s [94] and is now widely found in fish populations throughout North America, Australia, South Africa, and Europe [95]. Outbreaks of ERM usually begin with low mortalities which slowly escalate and may result in high losses. The problem may become large-scaled if chronically infected fish are exposed to stressful conditions such as high stocking densities and poor water quality [96]. *Y. ruckeri *is a nonspore-forming bacterium which does not possess a capsule, but often has a flagellum [97].

Historically, *Y. ruckeri *is fairly homogenous in biochemical reactions. However, *Y. ruckeri *strains have recently been grouped into clonal types on the basis of biotype, serotype, and outer membrane protein (OMP) profiles [98]. Strains of serovars I and II [99], equivalent to serotypes O1a and O2b, respectively [100], cause most epizootic outbreaks in cultured salmonids, serovar I being predominant in rainbow trout [101]. Within serovar I, six clonal OMP types have been recognized, but only two are associated with major disease outbreaks: clonal group 5, which includes the so-called Hagerman strain and clonal group 2 [98, 102]. Clonal group 5 comprises the majority of isolates, all of them motile and with a widespread distribution (Europe, North America, and South Africa). Clonal group 2 includes only nonmotile strains isolated in the UK.

More recently, multilocus sequence typing has revealed distinct phylogenetic divergence of *Y. ruckeri* from the rest of the *Yersinia* genus raising doubts about its taxonomic position [103]. This view has gained credibility after the genome sequencing of *Y. ruckeri*, which has a substantially reduced total genome size (3.58 to 3.89 Mb), compared with the 4.6 to 4.8 Mb seen in the genus generally [104]. In addition, *Y. ruckeri* was found to be the most evolutionarily distant member of the genus with a number of features distinct from other members of the genus.

Several common *Yersinia* genes were missing in *Y. ruckeri*. These included genes involved in xylose utilization, urease activity, B12-related metabolism, and the *mtnKADCBEU* gene cluster that comprises the majority of the methionine salvage pathway [104]. The genomic reduction achieved by losing these and other genes is suggestive of its means of adaptation to an obligatory life style in fish hosts.

## 8. *Renibacterium salmoninarum*

*Renibacterium salmoninarum* is a small Gram-positive diplobacillus, and the causative agent of bacterial kidney disease (BKD), which is a slowly progressive, systemic infection in salmonid fishes with a protracted course and an insidious nature [105]. The pathogen can be transmitted from fish to fish [106] or from adults to their progeny via eggs [107]. Infected fish may take months to show signs of disease. bacterial kidney disease is one of the most difficult bacterial diseases of fish to treat [108], mainly due to its ability to evade phagocytosis and invade and survive in host cells [109, 110]. *R. salmoninarum* is very slow growing, and it is extremely difficult to apply genetic manipulation techniques to study its gene functions.

*R. salmoninarum*, despite being an obligate intracellular pathogen of fish, is phylogenetically closest to the non-pathogenic environmental *Arthrobacter* species [51]. Based on 16S rRNA phylogenetic analysis, *R. salmoninarum* has been included in the actinomycetes subdivision and was found related to a subgroup harboring morphologically and chemotaxonomically rather heterogeneous taxa, including *Arthrobacter, Micrococcus, Cellulomonas, Jonesia, Promicromonospora, Stomatococcus*, and *Brevibacterium *[111]. In fact, *Arthrobacter davidanieli *is commercially used as a vaccine (commercially known as Renogen) and can provide significant cross-protection in Atlantic salmon, though not in Pacific salmon [112]. The genome sequencing of *R. salmoninarum* ATCC 33209 strain and two *Arthrobacter *strains, the TC1 and FB24, has revealed many interesting aspects of how this obligates fish pathogen evolved, via genomic reduction and horizontal gene acquisition, from members of the nonpathogenic genus *Arthrobacter* [51, 113]. A total of 1562 ORF clusters were similar in *R. salmoninarum *and *Arthrobacter *spp. demonstrating the genetic basis for the efficiency and cross-protection of the *A. davidanieli* vaccine.

There is significant genome reduction in *R. salmoninarum *genome, which is 1.44 Mb smaller than the chromosome of TC1 and 1.55 Mb smaller than the chromosome of FB24. The two *Arthrobacter *strains have several large plasmids that are not present in the ATCC 33209 strain. In addition, these plasmids do not have high levels of similarity to sequences in the *R. salmoninarum *chromosome [51].

The presence of many IS elements, pseudogenes, and genomic islands in *R. salmoninarum *genome coupled with a lack of restriction-modification systems contribute to the extensive disruption of ORFs as a strategy to reduce many pathways in the bacteria. Moreover, the highly homogeneous nature of *R. salmoninarum *with respect to the overall genomic structure, biochemical properties, and surface antigens [114, 115] points to the evolution of this pathogen towards a strictly intracellular life style.

Several virulence factors including capsular synthesis genes, heme acquisition operons, genes encoding possible hemolysins, and the poorly characterized *msa *genes identified in the *R. salmoninarum* genome seems to be horizontally acquired. *Arthrobacter *spp. lacks most of these gene sequences, thus underlining the differential evolution and adaptation of these two very closely related species to contrasting ecological niches.

## 9. *Streptococcus and Lactococcus*

Gram-positive cocci belonging to the genera *Streptococcus and Lactococcus* are increasingly being recognized as important fish pathogens all over the world [116]. There are several different species of Gram-positive cocci, including *Streptococcus parauberis*, *S. iniae*, *S. agalactiae* (syn. *Streptococcus difficilis*)*, S. phocae* [117, 118], *Lactococcus garvieae *(syn. *Enterococcus seriolicida)* [119], *L. piscium* [120–123], *Vagococcus salmoninarum*, and *Carnobacterium piscicola *[124], implicated in infectious diseases of warm water as well as cold water fishes.

Streptococcosis appears to have very few limitations in regard to geographic boundaries or host range, with outbreaks occurring in aquaculture facilities worldwide and in many different cultured species.* S. iniae, S.parauberis*, *S. agalactiae*, and *L. garvieae *are known as the major pathogens of streptococcosis and lactococcosis in *Oncorhynchus mykiss*, *Seriola quinqueradiata, Siganus canaliculatus*, and *Tilapia* spp. [125].* Recently, S. iniae* and *L. garvieae* are also recognized as emerging zoonotic pathogens, causing diseases in both fish and human beings [23, 126].

*S. iniae* is a *β*-haemolytic, Gram-positive coccus that causes generalized septicaemia and meningoencephalitis in a variety of warm water fishes [127], whereas *S. parauberis *is an *α*-hemolytic, Gram-positive coccus, mainly pathogenic in cultured turbot (*Scophthalmus maximus*) and olive flounder, *Paralichthys olivaceus*.* L. garvieae* causes a hyperacute and haemorrhagic septicemia in fishes particularly during the summer time. General pathological symptoms of streptococcosis and lactococcosis in fishes are hemorrhage, congestion, lethargy, dark pigmentation, erratic swimming, and exophthalmos with clouding of the cornea [117, 128].

Complete genome sequences of different strains of *S. parauberis* and *L. garvieae*, important pathogenic species isolated from both fish and human, have been published [129–132].

*S. parauberis *is recognized as the dominant etiological agent of streptococcosis in fish [117], whereas both *S. parauberis *and *S. uberis* are involved the causation of bovine mastitis in dairy cow [133, 134].

*S. parauberis *is closer to *S. uberis *than with other *Streptococcus *spp. and is biochemically and serologically indistinguishable from *S. uberis* [135]. Both species were earlier considered as type I and II of *S. uberis*, but later shown to be phylogenetically distinct and renamed the type I as *S. uberis* and type II as *S. parauberis* [134].

The *S. parauberis *strain KCTC11537BP genome size falls in the middle of the 1.8 to 2.3 Mb range of streptococcal genomes sequenced to date and the average G+C content of 35.6% is significantly lower than those of *S. pyogenes* [132]. About 78% of genes are shared between the genomes of *S. parauberis *strain KCTC11537BP and *S. uberis *NC_012004, but they differ significantly at two regions of the genome, demonstrating the genomic basis for their separation into two species.

*S. parauberis *genome encodes an M-like protein of *S. iniae *(SiM), which is an important virulence factor in *S. iniae* [136]. It also encodes *has*A and *has*B genes that may be involved in capsule production for resistance against phagocytosis. The genome analysis indicates that *S. parauberis *could possibly possess the ability to regulate the metabolism of more carbohydrates than other *Streptococcus *species and to synthesize all the aminoacids and regulatory factors required to adapt and survive in a highly hostile host environment.

Complete genome sequences of *L. garvieae *strain UNIUD074, isolated from diseased rainbow trout in Italy, a virulent strain Lg2 (serotype KG2) and a nonvirulent strain ATCC 49156 (serotype KG+), both isolated from diseased yellowtail in Japan have recently been published [130, 131]. In addition, genome sequence of *L. garvieae *strain 21881, isolated from a man suffering from septicemia has been published [129].

The strains Lg2 and ATCC 49156 have 99% sequence identity and share 1944 orthologous genes, but are different in 24 Lg2-specific genes that were absent in the ATCC 49156 genome. One of the Lg2-specific genes is a 16.5 kb capsule gene cluster, which confirms the earlier transmission electron microscopic finding that Lg2 is encapsulated, and ATCC 49156 is nonencapsulated [137]. In fact, the capsule gene cluster has the features of a horizontally acquired genomic island conferring virulence to the Lg2 strain but might have been lost from the ATCC 49156 strain while subculturing in the laboratory [131]. Both genomes carried three types of IS elements, prophage sequences, and integrase genes and were found smaller than those of at least five sequenced *L. lactis* genomes. The Lg2 genome lacks several aminoacid biosynthesis genes, which is a characteristic feature of pathogenic bacteria with reduced genomes. The Lg2 strain contains hemolysins, NADH oxidase and superoxide dismutase (SOD), adhesins and sortase, which are known virulence factors [137–139]. It also encodes a gene for phosphoglucomutase, a virulence factor conferring the resistance to peptide antimicrobials in *S. iniae* [140].

Although *L. garvieae* and *L. lactis* genomes share 75% CDS, about 25% genes are Lg2-specific hypothetical proteins and proteins of unknown functions, which may be involved in the virulence of the Lg2 strain. These findings indicate that *L. garvieae* and *L. lactis* have significantly diverged from the common ancestor, and the *L. garvieae *is evolving into a pathogenic species equipped with virulence features suitable for living in the host environment.

## 10. Mycobacteria

Chronic infections in fish caused by different species of mycobacteria have been well recognized [23, 141, 142]. Several slow growing as well as fast growing species of mycobacteria such as *Mycobacterium marinum*, *M. fortuitum*, *M. chelonae*, and *M. avium* have been isolated from wild and cultured fish suffering from mycobacteriosis in different parts of the world [143–145]. Among them, *M. marinum* is the most important fish pathogen, frequently isolated from a variety of fish species with granulomas [146]. It is also a known zoonotic pathogen, transmitted to man though fish handling in aquariums and aquaculture tanks, producing superficial and self-limiting lesions called “fish tank or aquarium tank granuloma” involving the cooler parts of the body such as hands, forearms, elbows, and knees [147, 148]. Although strain variation has been reported [149], there is significant intraspecies sequence homogeneity among different *M. mrinum* strains [150]. However, it is hypothesized that only certain strains of *M. marinum* have zoonotic potential [151]. Phylogenetic studies have shown that *M. marinum *is most closely related to *M. ulcerans *followed by *M. tuberculosis* [150]. Owing to this, *M. marinum* and *M. tuberculosis* share many virulence factors and significant pathological features and respond to similar antibiotics [152, 153]. Hence, *M. marinum* is also an important model organism to study the pathogenesis of tuberculosis [152, 153].

Interestingly, the genome of *M. marinum *is 50% bigger than that of *M. tuberculosis* and seems to have acquired a number of genes encoding NRPSs and the huge repertoire of PE, PPE, and ESX systems probably by HGT [154]. Both species might have evolved differently from a common environmental mycobacteria. *M. tuberculosis* might have adapted to its host intracellular life by extensive genome reduction and *M. marinum*, by and large retained or obtained genes required for its dual lifestyle and broad-host range.

## 11. Genome Sequencing to Find Novel Vaccine and Drug Targets in Fish Pathogens

Our understanding of the molecular basis of virulence of certain well-studied fish bacterial pathogens has increased dramatically during the past decade. This has resulted from the application of recombinant DNA technology and cell biology to investigate bacterial infections, and the development of genetic techniques for identifying virulence genes.

More recently, genome sequence information of several bacterial fish pathogens has become available from genome sequencing projects. There is strong reason to believe that this understanding will be exploited to develop new interventions against fish bacterial infections.

The relevance of sequencing projects for drug and vaccine discovery is obvious. During the “pregenomic” era, the vaccine candidate genes were individually identified by tedious gene knockout studies and virulence attenuation. But now, the complete genome sequencing provides information on every virulence gene and all potential vaccine candidates, and the sequence databases will become indispensable for research in fish vaccinology and drug development.

After sequencing, the open reading frames (ORFs) are searched against available databases for sequence similarity with genes of known functions in other organisms. There are several strategies for gene annotation employing the tools of predictive bioinformatics programs combined with analyses of the published literature.

Multiple target vaccine candidate genes can be chosen and deleted simultaneously by various strategies including global transposon mutagenesis and gene replacement techniques [155, 156] to study their effect on virulence and essentiality. A number of important virulence determinants identified in the sequenced genome can be targeted. For example, the sortase enzyme in Gram-positive fish pathogens would be a very attractive universal vaccine and therapeutic drug target, as it mediates covalent anchoring of many surface displayed antigenic and/or virulence related proteins in Gram-positive bacteria [139]. The inactivation or inhibition of the sortase enzyme can simultaneously prevent the surface display of a number of virulence factors, thus effectively attenuating the virulence of the pathogen [110, 157].

The availability of sequences of the complete surface antigenic repertoire of pathogens, including protein and noprotein antigens would facilitate strategies for rational design of vaccines and drugs. In addition, the recent availability of large collections of the “virulogenome” of fish bacterial pathogens will provide enormous virulence sequence information for DNA vaccination studies. The whole complement of IS elements, prophages, and pathogenicity islands that can harbor virulence, and antimicrobial resistance gene clusters can be easily identified in the genomes. The comparison of genomes of different strains of the same bacteria or closely related species can reveal how these strains or species behave differently while infecting fish hosts, thus opening exciting opportunities for functional genomic analysis of infection processes and pathogenesis. However, experimental validation of predicted functions of genes identified from sequencing projects has lagged far behind the speed of annotation, and the major challenge of researchers in the field today is to understand the functional framework of the sequenced genomes.

## 12. Conclusions

There has been a steady increase in the number of species of bacteria implicated in fish diseases. The common fish pathogenic bacterial species belong to the genera *Vibrio, Aeromonas, Flavobacterium, Yersinia, Edwardsiella, Streptococcus, lactococcus, Renibacterium*,* and Mycobacterium* [23]. However, there is growing indications that the pathogenic species spectrum as well as the geographic and host range is widening among fish pathogens [158–161], leading to the emergence of new pathogens. Unlike the situation in human and animal medicine, fish diseases pose unique and daunting challenges. Fish are always bathed in a continuous medium of water, and fish disease treatment is essentially a population medicine. In addition, the current treatment methods are largely ineffective, and the biology and genetics of most fish bacterial pathogens are poorly understood, limiting the application of modern science-based pathogen intervention strategies.

Rapid growth and expansion of genome sequencing of human and animal pathogens enabled better understanding of their biology, evolution, and host adaptation strategies, and helped in combating many major diseases. Unfortunately, such developments and progress in the genomics and functional genomics of fish pathogenic bacteria have been very slow. However, recent availability of cost-effective high-throughput sequencing technologies has set the pace of sequencing of more fish pathogenic bacteria. Genome sequencing of a number of important bacterial pathogens of fish has helped us to better understand their biology and genetics. The sequencing projects have unearthed exciting new information on the adaptive evolution of fish pathogens, for example, how the nonpathogenic and ubiquitous soil bacteria such as *Arthrobacter* sp. has evolved into a strictly obligate fish pathogen, *R. salmoninarum*, by shedding functional genes through genomic reduction to lead to a very cosy intracellular life style.

On the other hand, phenotypically similar strains of the same species differ in certain set of virulence gene clusters, acquired through HGT and become highly virulent. The capsule gene cluster in the* L. garvieae* Lg2 strain confers virulence compared to noncapsulated ATCC 49156, which lacks the gene cluster. Nonpathogenic strains acquire genomic islands from distantly related pathogenic species and emerge as new pathogens of fish.

Comparative pathogenomics of closely related bacteria has increased our knowledge of how they vary in their virulence and their ability to adapt to different ecological niches. This is clearly evident in the difference in virulence of various strains of *V. anguillarum* and *V. vulnificus,* and among the closely related species of the genus *Flavobacterium*. As more strain-specific sequence information on bacterial pathogens of fish becomes available, we will have a better understanding of the subtle genomic differences among strains with varying virulence characteristics.

The typical pathogen evolutionary strategy of acquiring, shuffling and shedding genes mediated by IS elements, pseudogenes, prophage sequences, and HGT is also observed in most bacterial pathogens of fish. It is certain that the new genomic information will bring paradigm changes in bacterial pathogenesis and should provide new perspectives to our current thinking on the evolutionary and adaptive strategies of aquatic bacteria and how they colonize and establish in wider ecological niches and new host species. Moreover, the identification of key virulence factors in pathogenic strains should help us design efficient drugs and vaccines to combat major bacterial pathogens of fish.

However, it should be stressed that the genomic information will provide only a snapshot of the microorganism. Highly virulent clones armed with one or more acquired virulence factors can suddenly develop from the existing harmless microorganisms in the face of environmental, antibiotic, and host-induced selective pressures.

More intriguingly, about 40% of the genes in sequenced bacterial genomes constitute new putative genes and hypothetical proteins with mysterious functions and are conserved among several different species of bacteria. Even in *Escherichia coli, *the most studied of all bacteria, only 54% genes have currently been functionally characterized based on experimental evidence [162]. A close scrutiny of the sequenced genomes of fish pathogens reveals that the above situation is essentially true for these pathogens as well. Although current advances in functional genomics, structural genomics and bioinformatics have contributed immensely to deciphering and extracting useful biological information from the vast genomic data, understanding and assigning functionality to the unique and new gene sequences discovered in the genomes will be the major task of genome biologists in the coming years.

## Figures and Tables

**Table 1:** Currently sequenced genomes of bacterial pathogens of fish.

Organisms	Size (Mb)	CDS**	Unknown/ Hypothetical genes (%)	Pseudogenes prophages, ISE/GEI	% GC	Chromosomes	Plasmids
*Vibrio anguillarum* 775 serotype O1	4.117	3880	26	92	44.3	2	1
*Vibrio anguillarum* 96F serotype O1	4.065	3766	26	38	42	2	0
*Vibrio anguillarum* RV22 serotype O2*β*	4.022	3949	26	68	43.1	2	0
*Vibrio ordalii* ATCC 33509	3.415	3281	—	31	43.3	2	0
*Vibrio harveyi* ATCC BAA-1116*	6.054	—	—	—	45.4	2	1
*Vibrio vulnificus* YJ016 biotype 1	5.26	5028	34	—	46.1	2	1
*Vibrio splendidus* strain LGP32	4.974	4498	24.8	—	43.8	2	0
*Aliivibrio salmonicida *strain LFI1238	4.655	4286	—	1179	38.3	2	4
*Flavobacterium psychrophilum* JIP02/86	2.862	2432	45.3	94	32.5	1	1
*Flavobacterium branchiophilum* FL-15	3.56	2867	—	54	32.9	1	—
*Flavobacterium columnare* ATCC 49512*	3.2	2896	—	—	32.0	1	—
*Edwardsiella tarda* EIB202	3.76	3486	28	97	59.7	1	1
*Edwardsiella ictaluri* 93–146*	3.812	3783	—	100	57.4	1	—
*Aeromonas hydrophila* ATCC 7966	4.744	5195	27.7	7	61.5	1	0
*Aeromonas salmonicida* A449	4.702	4437	—	258	58.5	1	5
*Aeromonas veronii *Strain B565	4.551	4057	—	—	58.7	1	—
*A. caviae *Ae398	4.43	—	—	6	61.4	1	1
*Renibacterium salmoninarum* ATCC 33209	3.155	3507	25.3	151	56.3	1	0
*Streptococcus parauberis*	2.143	2641	21.3	—	35.6	1	0
*Lactococcus garvieae* UNIUD074	2.172	2101	21.8	224	38.7	1	0
*Mycobacterium marinum* M	6.636	5424	26	65	62.5	1	1

* Unpublished.

** Coding sequences.

**Table 2:** Major bacterial pathogens of economically important fish.

Causative agent/species	Disease	Main host fish
Gram-negatives		
*Vibrio anguillarum*	Vibriosis	Salmonids, turbot, sea bass, striped bass, eel, ayu, cod, and red sea bream
*Aliivibrio salmonicida * (formerly *Vibrio salmonicida*)	Vibriosis	Atlantic salmon, cod
*Vibrio vulnificus*	Vibriosis	Eels, tilapia
*Vibrio ordalii*	Vibriosis	Salmonids
*Vibrio carchariae * (syn.: *Vibrio harveyi*)	Vibriosis, infectious gastroenteritis	Shark, abalone, red drum, sea bream, sea bass, cobia, and flounder
*Moritella viscosa * (formerly* Vibrio viscosus*)	Winter ulcer	Atlantic salmon
*Photobacterium damselae subsp. piscicida *(formerly* Pasteurella piscicida*)	Photobacteriosis (pasteurellosis)	Sea bream, sea bass, sole, striped bass, and yellowtail
*Pasteurella skyensis*	Pasteurellosis	Salmonids and turbot
*Tenacibaculum maritimum * (formerly* Flexibacter maritimus*)	Flexibacteriosis	Turbot, salmonids, sole, sea bass, gilthead sea bream, red sea bream, and flounder
*Flavobacterium psychrophilum*	Coldwater disease	Salmonids, carp, eel, tench, perch, ayu
* Flavobacterium branchiophila*	Bacterial gill disease	A broad range of cultured cold water and warm water salmonid and nonsalmonid fishes
*Flavobacterium columnare*	Columnaris disease	cyprinids, salmonids, silurids, eel, and sturgeon
*Pseudomonas anguilliseptica*	Pseudomonadiasis, winter disease	Sea bream, eel, turbot, and ayu
*Aeromonas salmonicida*	Furunculosis	salmon, trout, goldfish, koi and a variety of other fish species
* Aeromonas hydrophila * *Aeromonas veronii *Biovar Sobria *Aeromonas sobria *Biovar Sobria (Motile aeromonads)	Motile aeromonas septicemia (MAS), hemorrhagic septicemia, ulcer disease or red-sore disease, and epizootic ulcerative syndrome (EUS)	A wide variety of salmonid and nonsalmonid fish, sturgeon, tilapia, catfish, striped bass, and eel
*Edwardsiella ictaluri*	Enteric septicemia	Catfish and tilapia
*Edwardsiella tarda*	Edwardsiellosis	Salmon, carps, tilapia, catfish, striped bass, flounder, and yellowtail
*Yersinia ruckeri*	Enteric redmouth	Salmonids, eel, minnows, sturgeon, and crustaceans
*Piscirickettsia salmonis*	Piscirickettsiosis	Salmonids

Gram-positives		
*Lactococcus garvieae * (formerly* Enterococcus seriolicida*)	Streptococcosis or lactococcosis	Yellowtail and eel
*Streptococcus iniae*	Streptococcosis	Yellowtail, flounder, sea bass, and barramundi
*Streptococcus parauberis*	Streptococcosis	Turbot
*Streptococcus phocae*	Streptococcosis	Atlantic salmon
*Renibacterium salmoninarum*	Bacterial kidney disease	Salmonids
*Mycobacterium marinum*	Mycobacteriosis	Sea bass, turbot, and Atlantic salmon

## References

[1] Rothberg JM, Leamon JH (2008). The development and impact of 454 sequencing. *Nature Biotechnology*.

[2] Zhang J, Chiodini R, Badr A, Zhang G (2011). The impact of next-generation sequencing on genomics. *Journal of Genetics and Genomics*.

[3] Pennisi E (1998). Genome data shake tree of life. *Science*.

[4] Doolittle WF (1999). Phylogenetic classification and the universal tree. *Science*.

[5] Doolittle WF (1999). Lateral genomics. *Trends in Cell Biology*.

[6] Pennisi E (1999). Is it time to uproot the tree of life?. *Science*.

[7] Doolittle WF (2000). Uprooting the tree of life. *Scientific American*.

[8] Koonin EV, Makarova KS, Aravind L (2001). Horizontal gene transfer in prokaryotes: quantification and classification. *Annual Review of Microbiology*.

[9] Moran NA (2002). Microbial minimalism: genome reduction in bacterial pathogens. *Cell*.

[10] Mira A, Klasson L, Andersson SGE (2002). Microbial genome evolution: sources of variability. *Current Opinion in Microbiology*.

[11] Holm L (1986). Codon usage and gene expression. *Nucleic Acids Research*.

[12] Frost LS, Leplae R, Summers AO, Toussaint A (2005). Mobile genetic elements: the agents of open source evolution. *Nature Reviews Microbiology*.

[13] Dobrindt U, Hochhut B, Hentschel U, Hacker J (2004). Genomic islands in pathogenic and environmental microorganisms. *Nature Reviews Microbiology*.

[14] Dobrindt U, Hacker J, Sansonetti P (2010). How bacterial pathogens were constructed. *Bacterial Virulence: Basic Principles, Models and Global Approaches*.

[15] Pallen MJ, Wren BW (2007). Bacterial pathogenomics. *Nature*.

[16] Lawrence JG, Roth JR, Charlebois RL (1999). Genomic flux: genome evolution by gene loss and acquisition. *Organization of the Prokaryotic Genome*.

[17] Meyer FP (1991). Aquaculture disease and health management.. *Journal of Animal Science*.

[18] Harvell CD, Kim K, Burkholder JM (1999). Emerging marine diseases—climate links and anthropogenic factors. *Science*.

[19] Subasinghe R (1997). Fish health and quarantine; review of the state of the World aquaculture. *FAO Fisheries Circular*.

[20] Hjeltnes B, Roberts RJ, Roberts RJ, Bromag NR, Inglis V (1993). Vibriosis. *Bacterial Diseases of Fish*.

[21] Lightner DV, Redman RM (1998). Shrimp diseases and current diagnostic methods. *Aquaculture*.

[22] Bergh O, Nilsen F, Samuelsen OB (2001). Diseases, prophylaxis and treatment of the Atlantic halibut *Hippoglossus hippoglossus*: a review. *Diseases of Aquatic Organisms*.

[23] Austin B, Austin DA (1999). *Bacterial Fish Pathogens: Disease in Farmed and Wild Fish*.

[24] Austin B, Zhang XH (2006). *Vibrio harveyi*: a significant pathogen of marine vertebrates and invertebrates. *Letters in Applied Microbiology*.

[25] Amaro C, Biosca EG, Esteve C (1992). Comparative study of phenotypic and virulence properties in *Vibrio vulnificus* biotypes 1 and 2 obtained from a European eel farm experiencing mortalities. *Diseases of Aquatic Organisms*.

[26] Biosca EG, Llorens H, Garay E, Amaro C (1993). Presence of a capsule in *Vibrio vulnificus* biotype 2 and its relationship to virulence for eels. *Infection and Immunity*.

[27] Benediktsdóttir E, Verdonck L, Spröer C, Helgason S, Swings J (2000). Characterization of *Vibrio viscosus* and *Vibrio wodanis* isolated at different geographical locations: a proposal for reclassification of *Vibrio viscosus* as *Moritella viscosa* comb. nov.. *International Journal of Systematic and Evolutionary Microbiology*.

[28] Actis LA, Tolmasky ME, Crosa JH, Woo PTK, Bruno DW (2011). Vibriosis. *Fish Diseases and Disorders, Viral, Bacterial, and Fungal Infections*.

[29] Hjerde E, Lorentzen M, Holden MTG (2008). The genome sequence of the fish pathogen *Aliivibrio salmonicida* strain LFI1238 shows extensive evidence of gene decay. *BMC Genomics*.

[30] Gulig PA, Crécy-Lagard VD, Wright AC, Walts B, Telonis-Scott M, McIntyre LM (2010). SOLiD sequencing of four *Vibrio vulnificus* genomes enables comparative genomic analysis and identification of candidate clade-specific virulence genes. *BMC Genomics*.

[31] Naka H, Dias GM, Thompson CC, Dubay C, Thompson FL, Crosa JH (2011). Complete genome sequence of the marine fish pathogen *Vibrio anguillarum* harboring the pJM1 virulence plasmid and genomic comparison with other virulent strains of *V. anguillarum* and *V. ordalii*. *Infection and Immunity*.

[32] Larsen JL, Pedersen K, Dalsgaard I (1994). *Vibrio anguillarum* serovars associated with vibriosis in fish. *Journal of Fish Diseases*.

[33] Sorensen UBS, Larsen JL (1986). Serotyping of *Vibrio anguillarum*. *Applied and Environmental Microbiology*.

[34] Toranzo AE, Barja JL (1990). A review of the taxonomy and seroepizootiology of *Vibrio anguillarum*, with special reference to aquaculture in the NorthWest Spain. *Diseases of Aquatic Organisms*.

[35] Crosa JH (1980). A plasmid associated with virulence in the marine fish pathogen *Vibrio anguillarum* specifies an iron-sequestering system. *Nature*.

[36] Toranzo AE, Barja JL, Potter SA (1983). Molecular factors associated with virulence of marine vibrios isolated from striped bass in Chesapeake Bay. *Infection and Immunity*.

[37] Schiewe MH, Trust TJ, Crosa JH (1981). *Vibrio ordalii* sp. nov.: a causative agent of vibriosis in fish. *Current Microbiology*.

[38] Ransom DP, Lannan CN, Rohovec JS, Fryer JL (1984). Comparison of histopathology caused by *Vibrio anguillarum* and *Vibrio ordalii* in three species of Pacific salmon. *Journal of Fish Diseases*.

[39] Makino K, Oshima K, Kurokawa K (2003). Genome sequence of *Vibrio parahaemolyticus*: a pathogenic mechanism distinct from that of *V. cholerae*. *The Lancet*.

[40] Okada N, Matsuda S, Matsuyama J (2010). Presence of genes for type III secretion system 2 in *Vibrio mimicus* strains. *BMC Microbiology*.

[41] Alam A, Miller KA, Chaand M, Butler JS, Dziejman M (2011). Identification of *Vibrio cholerae* type III secretion system effector proteins. *Infection and Immunity*.

[42] Osorio CR, Juiz-Rio S, Lemos ML (2006). A siderophore biosynthesis gene cluster from the fish pathogen *Photobacterium damselae* subsp. *piscicida* is structurally and functionally related to the *Yersinia* high-pathogenicity island. *Microbiology*.

[43] Yip ES, Grublesky BT, Hussa EA, Visick KL (2005). A novel, conserved cluster of genes promotes symbiotic colonization and *σ*-dependent biofilm formation by *Vibrio fischeri*. *Molecular Microbiology*.

[44] Kim HS, Park SJ, Lee KH (2009). Role of NtrC-regulated exopolysaccharides in the biofilm formation and pathogenic interaction of *Vibrio vulnificus*. *Molecular Microbiology*.

[45] Sanjuan E, Fouz B, Oliver JD, Amaro C (2009). Evaluation of genotypic and phenotypic methods to distinguish clinical from environmental *Vibrio vulnificus* strains. *Applied and Environmental Microbiology*.

[46] Bisharat N, Cohen DI, Maiden MC, Crook DW, Peto T, Harding RM (2007). The evolution of genetic structure in the marine pathogen, *Vibrio vulnificus*. *Infection, Genetics and Evolution*.

[47] Schrøder MB, Espelid S, Jørgensen TØ (1992). Two serotypes of *Vibrio salmonicida* isolated from diseased cod (*Gadus morhua* L.); virulence, immunological studies and vaccination experiments. *Fish and Shellfish Immunology*.

[48] Tomich M, Planet PJ, Figurski DH (2007). The tad locus: postcards from the widespread colonization island. *Nature Reviews Microbiology*.

[49] Siguier P, Filee J, Chandler M (2006). Insertion sequences in prokaryotic genomes. *Current Opinion in Microbiology*.

[50] Parkhill J, Dougan G, James KD (2001). Complete genome sequence of a multiple drug resistant *Salmonella enterica* serovar Typhi CT18. *Nature*.

[51] Wiens GD, Rockey DD, Wu Z (2008). Genome sequence of the fish pathogen *Renibacterium salmoninarum* suggests reductive evolution away from an environmental *Arthrobacter* ancestor. *Journal of Bacteriology*.

[52] Martin-Carnahan A, Joseph SW, Garrity GM (2005). Aeromonadaceae. *Bergey’s Manual of Systematic Bacteriology*.

[53] Figueras MJ (2005). Clinical relevance of *Aeromonas* sM503. *Reviews in Medical Microbiology*.

[54] Seshadri R, Joseph SW, Chopra AK (2006). Genome sequence of *Aeromonas hydrophila* ATCC 7966T: jack of all trades. *Journal of Bacteriology*.

[55] Reith ME, Singh RK, Curtis B (2008). The genome of *Aeromonas salmonicida* subsp. *salmonicida* A449: Insights into the evolution of a fish pathogen. *BMC Genomics*.

[56] Li Y, Liu Y, Zhou Z (2011). Complete genome sequence of *Aeromonas veronii* strain B565. *Journal of Bacteriology*.

[57] Beatson SA, de Luna MDG, Bachmann NL (2011). Genome sequence of the emerging pathogen *Aeromonas caviae*. *Journal of Bacteriology*.

[58] Bernoth EM, Bernoth EM, Ellis AE, Midtlyng PJ, Olivier G, Smith P (1997). Furunculosis: the history of the diseases and of disease research. *Furunculosis, Multidisciplinary Fish Disease Research*.

[59] Hiney M, Olivier G, Woo PTK, Bruno DW (1999). Furunculosis (*Aeromonas salmonicida*). *Fish Diseases and Disorders III: Viral, Bacterial and Fungal Infections*.

[60] Paniagua C, Rivero O, Anguita J, Naharro G (1990). Pathogenicity factors and virulence for rainbow trout (*Salmo gairdneri*) of motile *Aeromonas* spp. isolated from a river. *Journal of Clinical Microbiology*.

[61] Burr SE, Pugovkin D, Wahli T, Segner H, Frey J (2005). Attenuated virulence of an *Aeromonas salmonicida* subsp. *salmonicida* type III secretion mutant in a rainbow trout model. *Microbiology*.

[62] Sha J, Pillai L, Fadl AA, Galindo CL, Erova TE, Chopra AK (2005). The type III secretion system and cytotoxic enterotoxin alter the virulence of *Aeromonas hydrophila*. *Infection and Immunity*.

[63] Janda JM, Kokka RP (1991). The pathogenicity of *Aeromonas* strains relative to genospecies and phenospecies identification. *FEMS Microbiology Letters*.

[64] Madetoja J, Dalsgaard I, Wiklund T (2002). Occurrence of *Flavobacterium psychrophilum* in fish-farming environments. *Diseases of Aquatic Organisms*.

[65] Nematollahi A, Decostere A, Pasmans F, Haesebrouck F (2003). *Flavobacterium psychrophilum* infections in salmonid fish. *Journal of Fish Diseases*.

[66] Leahy JG, Colwell RR (1990). Microbial degradation of hydrocarbons in the environment. *Microbiological Reviews*.

[67] Lehmann J, Mock D, Stuerenberg FJ, Bernardet JF (1991). First isolation of *Cytophaga psychrophila* from a systemic disease in eel and cyprinids. *Diseases of Aquatic Organisms*.

[68] Holt RA (1987). *Cytophaga psychrophila, the causative agent of bacterial cold-water disease in salmonid fish*.

[69] Heo GJ, Kasai K, Wakabayashi H (1990). Occurrence of *Flavobacterium branchiophila* associated with bacterial gill disease at a trout hatchery. *Fish Pathology*.

[70] Ostland VE, Lumsden JS, MacPhee DD, Ferguson HW (1994). Characteristics of *Flavobacterium branchiophilum*, the cause of salmonid bacterial gill disease in Ontario. *Journal of Aquatic Animal Health*.

[71] Bernardet JF (1989). *Flexibacter columnaris*: first description in France and comparison with bacterial strains from other origins. *Diseases of Aquatic Organisms*.

[72] Speare DJ, Ferguson HW (1989). Clinical and pathological features of common gill diseases of cultured salmonids in Ontario. *Canadian Veterinary Journal*.

[73] Bernardet JF, Segers P, Vancanneyt M, Berthe F, Kersters K, Vandamme P (1996). Cutting a gordian knot: emended classification and description of the genus *Flavobacterium*, emended description of the family *Flavobacteriaceae*, and proposal of *Flavobacterium hydatis* nom. nov. (basonym, Cytophaga aquatilis Strohl and Tait 1978). *International Journal of Systematic Bacteriology*.

[74] Duchaud E, Boussaha M, Loux V (2007). Complete genome sequence of the fish pathogen *Flavobacterium psychrophilum*. *Nature Biotechnology*.

[75] Touchon M, Barbier P, Bernardet JF (2011). Complete genome sequence of the fish pathogen *Flavobacterium branchiophilum*. *Applied and Environmentl Microbiology*.

[76] Lawrence ML, Karsi A, Tekedar HC Comparative genomics of *Flavobacterium columnare* isolates from two genetic divisions and with different pathogenic potential for channel catfish (*Ictalurus punctatus*).

[77] Rodkhum C, Hirono I, Crosa JH, Aoki T (2005). Four novel hemolysin genes of *Vibrio anguillarum* and their virulence to rainbow trout. *Microbial Pathogenesis*.

[78] Palmer M (2001). The family of thiol-activated, cholesterol-binding cytolysins. *Toxicon*.

[79] Shoji M, Ratnayake DB, Shi Y (2002). Construction and characterization of a nonpigmented mutant of *Porphyromonas gingivalis*: Cell surface polysaccharide as an anchorage for gingipanis. *Microbiology*.

[80] Sato K, Sakai E, Veith PD (2005). Identification of a new membrane-associated protein that influences transport/maturation of gingipains and adhesins of *Porphyromonas gingivalis*. *The Journal of Biological Chemistry*.

[81] Abbott SL, Janda JM (2006). The genus *Edwardsiella*. *Prokaryotes*.

[82] Clarridge JE, Musher DM, Fanstein V, Wallace RJ (1980). Extra-intestinal human infection caused by *Edwardsiella tarda*. *Journal of Clinical Microbiology*.

[83] Nagel P, Serritella A, Layden TJ (1982). *Edwardsiella tarda* gastroenteritis associated with a pet turtle. *Gastroenterology*.

[84] Rogers WA, Anderson DP, Dorson M, Dubourget Ph (1983). Edwardsiellosis in fishes. *Antigens of Fish Pathogens. Les Antigenes des Microorganisms Pathogenes des Poissons*.

[85] Meyer FP, Bullock GL (1973). *Edwardsiella tarda*, a new pathogen of channel catfish (*Ictalurus punctatus*). *Applied Microbiology*.

[86] Ling SHM, Wang XH, Xie L, Lim TM, Leung KY (2000). Use of green fluorescent protein (GFP) to study the invasion pathways of *Edwardsiella tarda* in *in vivo* and *in vitro* fish models. *Microbiology*.

[87] Newton JC, Wolfe LG, Grizzle JM, Plumb JA (1989). Pathology of experimental enteric septicaemia in channel catfish, *Ictalurus punctatus* (Rafinesque), following immersion-exposure to *Edwardsiella ictaluri*. *Journal of Fish Diseases*.

[88] Wang Q, Yang M, Xiao J (2009). Genome sequence of the versatile fish pathogen *Edwardsiella tarda* provides insights into its adaptation to broad host ranges and intracellular niches. *PLoS One*.

[89] Williams ML, Gillaspy AF, Dyer DW (2012). Genome sequence of *Edwardsiella ictaluri* 93–146, a strain associated with a natural channel catfish outbreak of enteric septicemia of catfish. *Journal of Bacteriology*.

[90] Partridge SR, Hall RM (2003). The IS1111 family members IS4321 and IS5075 have sub-terminal inverted repeats and target the terminal inverted repeats of Tn21 family transposons. *Journal of Bacteriology*.

[91] Tanabe T, Funahashi T, Nakao H, Miyoshi SI, Shinoda S, Yamamoto S (2003). Identification and characterization of genes required for biosynthesis and transport of the siderophore vibrioferrin in *Vibrio parahaemolyticus*. *Journal of Bacteriology*.

[92] Wang Q, Liu Q, Ma Y, Zhou L, Zhang Y (2007). Isolation, sequencing and characterization of cluster genes involved in the biosynthesis and utilization of the siderophore of marine fish pathogen *Vibrio alginolyticus*. *Archives of Microbiology*.

[93] Furones MD, Rodgers CJ, Munn CB (1993). *Yersinia ruckeri*, the causal agent of enteric redmouth disease (ERM) in fish. *Annual Review of Fish Diseases*.

[94] Rucker RR (1966). Redmouth disease of rainbow trout (*Salmo gairdneri*). *Bulletin de l’Office International des Epizooties*.

[95] Tobback E, Decostere A, Hermans K, Haesebrouck F, Chiers K (2007). *Yersinia ruckeri* infections in salmonid fish. *Journal of Fish Diseases*.

[96] Horne MT, Barnes AC, Woo PTK, Bruno DW (1999). Enteric redmouth disease (*Y. ruckeri*). *Fish Diseases and Disorders: Viral, Bacterial and Fungal Infections*.

[97] Ross AJ, Rucker RR, Ewing WH (1966). Description of a bacterium associated with redmouth disease of rainbow trout (*Salmo gairdneri*). *Canadian Journal of Microbiology*.

[98] Davies RL (1991). Virulence and serum-resistance in different clonal groups and serotypes of *Yersinia ruckeri*. *Veterinary Microbiology*.

[99] Stevenson RMV, Airdrie DW (1984). Serological variation among *Yersinia ruckeri* strains. *Journal of Fish Diseases*.

[100] Romalde JL, Magarinos B, Barja JL, Toranzo AE (1993). Antigenic and molecular characterization of *Yersinia ruckeri* proposal for a new intraspecies classification. *Systematic and Applied Microbiology*.

[101] Stevenson RM (1997). Immunization with bacterial antigens: yersiniosis. *Developments in Biological Standardization*.

[102] Davies RL (1991). *Yersinia ruckeri* produces four iron-regulated proteins but does not produce detectable siderophores. *Journal of Fish Diseases*.

[103] Kotetishvili M, Kreger A, Wauters G, Morris JG, Sulakvelidze A, Stine OC (2005). Multilocus sequence typing for studying genetic relationships among *Yersinia* species. *Journal of Clinical Microbiology*.

[104] Chen PE, Cook C, Stewart AC (2010). Genomic characterization of the *Yersinia* genus. *Genome Biology*.

[105] Sanders JE, Fryer JL (1980). *Renibacterium salmoninarum* gen. nov., sp. nov., the causative agent of bacterial kidney disease in salmonid fishes. *International Journal of Systematic Bacteriology*.

[106] Mitchum DL, Sherman LE (1981). Transmission of bacterial kidney disease from wild to stocked hatchery trout. *Canadian Journal of Fisheries and Aquatic Sciences*.

[107] Bullock GL (1980). *Bacterial Kidney Disease of Salmonid Fishes Caused by Renibacterium salmoninarum*.

[108] Wolf KE, Dunbar CE (1959). Test of 34 therapeutic agents for control of kidney disease in trout. *Transactions of American Fisheries Society*.

[109] Gutenberger SK, Duimstra JR, Rohovec JS, Fryer JL (1997). Intracellular survival of *Renibacterium salmoninarum* in trout mononuclear phagocytes. *Disease of Aquatic Organisms*.

[110] Sudheesh PS, Crane S, Cain KD, Strom MS (2007). Sortase inhibitor phenyl vinyl sulfone inhibits *Renibacterium salmoninarum* adherence and invasion of host cells. *Diseases of Aquatic Organisms*.

[111] Stackebrandt E, Wehmeyer U, Nader H, Fiedler F (1988). Phylogenetic relationship of the fish pathogenic *Renibacterium salmoninarum* to *Arthrobacter*, *Micrococcus* and related taxa. *FEMS Microbiology Letters*.

[112] Salonius K, Siderakis C, MacKinnon AM, Griffiths SG (2005). Use of *Arthrobacter davidanieli* as a live vaccine against *Renibacterium salmoninarum* and *Piscirickettsia salmonis* in salmonids. *Developments in Biologicals*.

[113] Mongodin EF, Shapir N, Daugherty SC (2006). Secrets of soil survival revealed by the genome sequence of *Arthrobacter aurescens * TC1. *PLoS Genetics*.

[114] Getchell RG, Rohovec JS, Fryer JL (1985). Comparison of *Renibacterium salmoninarum* isolates by antigenic analysis. *Fish Pathology*.

[115] Fiedler F, Draxl R (1986). Biochemical and immunochemical properties of the cell surface of *Renibacterium salmoninarum*. *Journal of Bacteriology*.

[116] Kitao T, Inglis V, Roberts RJ, Bromage NR (1993). Streptococcal infections. *Bacterial Disease of Fish*.

[117] Domenech A, Fernandez-Garayzabal JF, Pasqual C (1996). Streptococcosis in cultured turbot, *Scophthalmus maximus* (L.), associated with *Streptococcus parauberis*. *Journal of Fish Diseases*.

[118] Romalde JL, Ravelo C, Valdés I (2008). *Streptococcus phocae*, an emerging pathogen for salmonid culture. *Veterinary Microbiology*.

[119] Vendrell D, Balcázar JL, Ruiz-Zarzuela I, de Blas I, Gironés O, Múzquiz JL (2006). *Lactococcus garvieae* in fish: a review. *Comparative Immunology, Microbiology and Infectious Diseases*.

[120] Collins MD, Farrow JAE, Phillips BA, Kandler O (1983). *Streptococcus garvieae* sp. nov. and *Streptococcus plantarum* sp. nov.. *Journal of General Microbiology*.

[121] Williams AM, Fryer JL, Collins MD (1990). *Lactococcus piscium* sp. nov. a new *Lactococcus* species from salmonid fish. *FEMS Microbiology Letters*.

[122] Domenech A, Prieta J, Fernandez-Garayzabal JF (1993). Phenotypic and phylogenetic evidence for a close relationship between *Lactococcus garvieae* and *Enterococcus seriolicida*. *Microbiologia SEM*.

[123] Eldar A, Ghittino C, Asanta L (1996). *Enterococcus seriolicida* is a junior synonym of *Lactococcus garvieae*, a causative agent of septicemia and meningoencephalitis in fish. *Current Microbiology*.

[124] Wallbanks S, Martinez-Murcia AJ, Fryer JL, Phillips BA, Collins MD (1990). 16S rRNA sequence determination for members of the genus *Carnobacterium* and related lactic acid bacteria and description of *Vagococcus salmoninarum* sp. nov.. *International Journal of Systematic Bacteriology*.

[125] Bromage ES, Owens L (2002). Infection of barramundi *Lates calcarifer* with *Streptococcus iniae*: effects of different routes of exposure. *Diseases of Aquatic Organisms*.

[126] Elliott JA, Collins MD, Pigott NE, Facklam RR (1991). Differentiation of *Lactococcus lactis* and *Lactococcus garvieae* from humans by comparison of whole-cell protein patterns. *Journal of Clinical Microbiology*.

[127] Perera RP, Johnson SK, Collins MD, Lewis DH (1994). *Streptococcus iniae* associated with mortality of *Tilapia nilotica* X *T.aurea* hybrids. *Journal of Aquatic Animal Health*.

[128] Kusuda R, Kawai K, Salati F, Banner CR, Fryer JL (1991). *Enterococcus seriolicida* sp. nov., a fish pathogen. *International Journal of Systematic Bacteriology*.

[129] Aguado-Urda M, López-Campos GH, Blanco MM (2011). Genome sequence of *Lactococcus garvieae* 21881, isolated in a case of human septicemia. *Journal of Bacteriology*.

[130] Reimundo P, Pignatelli M, Alcaraz LD, D’Auria G, Moya A, Guijarro JA (2011). Genome sequence of *Lactococcus garvieae* UNIUD074, isolated in Italy from a lactococcosis outbreak. *Journal of Bacteriology*.

[131] Morita H, Toh H, Oshima K (2011). Complete genome sequence and comparative analysis of the fish pathogen *Lactococcus garvieae*. *PLoS One*.

[132] Nho SW, Hikima JI, Cha IS (2011). Complete genome sequence and immunoproteomic analyses of the bacterial fish pathogen *Streptococcus parauberis*. *Journal of Bacteriology*.

[133] Watts JL (1988). Etiological agents of bovine mastitis. *Veterinary Microbiology*.

[134] Williams AM, Collins MD (1990). Molecular taxonomic studies on *Streptococcus uberis* types I and II. Description of *Streptococcus parauberis* sp. nov.. *Journal of Applied Bacteriology*.

[135] Bentley RW, Leigh JA, Collins MD (1991). Intrageneric structure of *Streptococcus* based on comparative analysis of small-subunit rRNA sequences. *International Journal of Systematic Bacteriology*.

[136] Locke JB, Aziz RK, Vicknair MR, Nizet V, Buchanan JT (2008). *Streptococcus iniae* M-like protein contributes to virulence in fish and is a target for live attenuated vaccine development. *PLoS One*.

[137] Kawanishi M, Yoshida T, Kijima M (2007). Characterization of *Lactococcus garvieae* isolated from radish and broccoli sprouts that exhibited a KG+ phenotype, lack of virulence and absence of a capsule. *Letters in Applied Microbiology*.

[138] Mitchell TJ (2003). The pathogenesis of streptococcal infections: from tooth decay to meningitis. *Nature Reviews. Microbiology*.

[139] Marraffini LA, Dedent AC, Schneewind O (2006). Sortases and the art of anchoring proteins to the envelopes of gram-positive bacteria. *Microbiology and Molecular Biology Reviews*.

[140] Buchanan JT, Stannard JA, Lauth X (2005). *Streptococcus iniae*phosphoglucomutase is a virulence factor and a target for vaccine development. *Infection and Immunity*.

[141] Frerichs GN, Inglis V, Roberts RJ, Bromage NR (1993). Acid-fast fish pathogens. *Bacterial Diseases of Fish*.

[142] Noga EJ (1996). *Fish disease diagnosis and treatment*.

[143] Wayne LG, Kubica GP, Sneath PHA, Mair NS, Sharpe ME, Holt JG (1986). Genus *Mycobacterium* Lehmann and Neumann 1896, 363AL. *Bergey’s Manual of Systematic Bacteriology*.

[144] Thoen CO, Schliesser TA, Kubica GP, Wayne LG (1984). Mycobacterial infections in cold-blooded animals. *The Mycobacteria*.

[145] Sanders GE, Swaim LE (2001). Atypical piscine Mycobacteriosis in Japanese medaka (*Oryzias latipes*). *Comparative Medicine*.

[146] Stoskopf MK (1993). *Fish Medicine*.

[147] Smith DT, Willett HP, Joklik K, Willet HP, Amos DB (1980). Other *Mycobacterium* species. *Zinsser Microbiology*.

[148] Huminer D, Pitlik SD, Block C (1986). Aquarium-borne *Mycobacterium marinum* skin infection. Report of a case and review of the literature. *Archives of Dermatology*.

[149] Ucko M, Colorni A, Kvitt H, Diamant A, Zlotkin A, Knibb WR (2002). Strain variation in *Mycobacterium marinum* fish isolates. *Applied and Environmental Microbiology*.

[150] Yip MJ, Porter JL, Fyfe JAM (2007). Evolution of *Mycobacterium ulcerans* and other mycolactone-producing mycobacteria from a common *Mycobacterium marinum* progenitor. *Journal of Bacteriology*.

[151] Ucko M, Colorni A (2005). *Mycobacterium marinum* infections in fish and humans in Israel. *Journal of Clinical Microbiology*.

[152] Volkman HE, Clay H, Beery D, Chang JCW, Sherman DR, Ramakrishnan L (2004). Tuberculous granuloma formation is enhanced by a *Mycobacterium* virulence determinant. *PLoS Biology*.

[153] Swaim LE, Connolly LE, Volkman HE, Humbert O, Born DE, Ramakrishnan L (2006). *Mycobacterium marinum* infection of adult zebrafish causes caseating granulomatous tuberculosis and is moderated by adaptive immunity. *Infection and Immunity*.

[154] Stinear TP, Seemann T, Harrison PF (2008). Insights from the complete genome sequence of *Mycobacterium marinum* on the evolution of *Mycobacterium tuberculosis*. *Genome Research*.

[155] McFadden J (1996). Recombination in mycobacteria. *Molecular Microbiology*.

[156] Salama NR, Shepherd B, Falkow S (2004). Global transposon mutagenesis and essential gene analysis of *Helicobacter pylori*. *Journal of Bacteriology*.

[157] Cossart P, Jonquières R (2000). Sortase, a universal target for therapeutic agents against Gram-positive bacteria?. *Proceedings of the National Academy of Sciences of the United States of America*.

[158] Maher M, Palmer R, Gannon F, Smith T (1995). Relationship of a novel bacterial fish pathogen to *Streptobacillus monilifomris* and the fusobacteria group, based on 16S ribosomal RNA analysis. *Systematic and Applied Microbiology*.

[159] Fryer JL, Mauel MJ (1997). The rickettsia: an emerging group of pathogens in fish. *Emerging Infectious Diseases*.

[160] Birkbeck TH, Laidler LA, Grant AN, Cox DI (2002). *Pasteurella skyensis* sp. nov., isolated from Atlantic salmon (*Salmo salar* L.). *International Journal of Systematic and Evolutionary Microbiology*.

[161] Michel C, Bernardet JF, Daniel P, Chilmonczyk S, Urdaci M, de Kinkelin P (2002). Clinical and aetiological aspects of a summer enteritic syndrome associated with the sporulating segmented filamentous bacterium “*Candidatus Arthromitus*” in farmed rainbow trout, *Oncorhynchus mykiss* (Walbaum). *Journal of Fish Diseases*.

[162] Riley M, Abe T, Arnaud MB (2006). *Escherichia coli* K-12: a cooperatively developed annotation snapshot—2005. *Nucleic Acids Research*.

